# Complete genome sequence of lovastatin producer *Aspergillus terreus* ATCC 20542 and evaluation of genomic diversity among *A. terreus* strains

**DOI:** 10.1007/s00253-021-11133-0

**Published:** 2021-01-30

**Authors:** Małgorzata Ryngajłło, Tomasz Boruta, Marcin Bizukojć

**Affiliations:** 1 Institute of Molecular and Industrial Biotechnology, Lodz University of Technology, B. Stefanowskiego 4/10, 90-924 Lodz, Poland; 2 Faculty of Process and Environmental Engineering, Department of Bioprocess Engineering, Lodz University of Technology, ul. Wolczanska 213, 90-924 Lodz, Poland

**Keywords:** *Aspergillus terreus*, Genome, Comparative genomics, Secondary metabolites

## Abstract

**Abstract:**

In the present study, the complete genome of a filamentous fungus *Aspergillus terreus* ATCC 20542 was sequenced, assembled, and annotated. This strain is mainly recognized for being a model wild-type lovastatin producer and a parental strain of high-yielding industrial mutants. It is also a microorganism with a rich repertoire of secondary metabolites that has been a subject of numerous bioprocess-related studies. In terms of continuity, the genomic sequence provided in this work is of the highest quality among all the publicly available genomes of *A. terreus* strains. The comparative analysis revealed considerable diversity with regard to the catalog of biosynthetic gene clusters found in *A. terreus*. Even though the cluster of lovastatin biosynthesis was found to be well-conserved at the species level, several unique genes putatively associated with metabolic functions were detected in *A. terreus* ATCC 20542 that were not detected in other investigated genomes. The analysis was conducted also in the context of the primary metabolic pathways (sugar catabolism, biomass degradation potential, organic acid production), where the visible differences in gene copy numbers were detected. However, the species-level genomic diversity of *A. terreus* was more evident for secondary metabolism than for the well-conserved primary metabolic pathways. The newly sequenced genome of *A. terreus* ATCC 20542 was found to harbor several unique sequences, which can be regarded as interesting subjects for future experimental efforts on *A. terreus* metabolism and fungal biosynthetic capabilities.

**Key points:**

*• The high-quality genome of Aspergillus terreus ATCC 20542 has been assembled and annotated.*

*• Comparative analysis with other sequenced Aspergillus terreus strains has revealed considerable diversity in biosynthetic gene repertoire, especially related to secondary metabolism.*

*• The unique genomic features of A. terreus ATCC 20542 are discussed.*

**Supplementary Information:**

The online version contains supplementary material available at 10.1007/s00253-021-11133-0.

## Introduction

*Aspergillus terreus* is a species associated mainly with three distinct scientific and engineering areas, namely, the invasive fungal infections (Lass-Flörl 2018; Pastor and Guarro 2014; Steinbach et al. 2004), the microbial production of itaconic acid, an important building block used in chemical industry (Klement and Buchs 2013; Krull et al. 2017; Okabe et al. 2009), and the biosynthesis of lovastatin, a secondary metabolite applied as a cholesterol-lowering drug in hypercholesterolemia treatments (Alberts et al. 1980; Barrios-González and Miranda 2010; Endo 2010; Hasan et al. 2019; Mulder et al. 2015). This microorganism is also considered to be a rich source of biotechnologically relevant enzymes, e.g., cellulases, xylanases, and lipases (Ghanem et al. 2000; Sethi et al. 2016; Sohail et al. 2016). Being a fungus of dual (i.e., harmful or useful) nature, extensively investigated both in the clinical and industrial contexts, *A. terreus* continues to serve as a subject of numerous research efforts (Vyas 2011).

The first complete genome sequence of *A. terreus*, specifically of the clinical strain *A. terreus* NIH2624, was provided by the BROAD Institute in 2005 and encompassed 29.36 megabase pairs (Mb). Even today, the genome of *A. terreus* NIH2624 remains a high-quality reference for genetic studies on *A. terreus*. It took 11 years for the next genome of this species (strain 45A, also referred to as KM017963) to be published (Savitha et al. 2016). Within the next 4 years, the genomes of strains w25, T3 Kankrej, TN-484 (Kanamasa et al. 2019), M6925 (Palanivel et al. 2020), and IFO6365 (Takahashi et al. 2020) were submitted to the NCBI database. Surprisingly, the genome of *A. terreus* ATCC 20542, a model lovastatin-producing microorganism deposited in the American Type Culture Collection, has never been released. Originally isolated from soil in Spain (Alberts et al. 1980), this wild-type strain was applied in numerous bioprocess-related studies focused on the optimization of lovastatin production (see the review of Mulder et al. 2015 and references therein). Importantly, it was used in the mutagenesis experiments as a parent of high-yielding industrial strains (Buckland et al. 1989; Hendrickson et al. 1999; Vinci et al. 1991) and employed in the pioneering research on the lovastatin biosynthetic gene cluster (Kennedy et al. 1999). Even though Microbia (the former name of Ironwood Pharmaceuticals, Inc, Boston, Massachusetts, USA), an industrial biotechnology company, did share the sequence data of *A. terreus* ATCC 20542 in relation with the transcriptomic and metabolomic experiments on lovastatin production (Askenazi et al. 2003), the genomic fragment that was made publicly available encompassed only 0.14 Mb of sequence and was far from being considered a completely sequenced genome.

If one aims to perform any system-level studies focused on elucidating the mechanisms of lovastatin biosynthesis, *A. terreus* ATCC 20542 should be regarded as a model strain and a highly recommended research subject. Having access to a high-quality, complete genome sequence is an essential prerequisite for an in-depth analysis of this kind, as well as for the efforts aimed at designing effective lovastatin-producing cell factories. In the context of uncovering the fungal biosynthetic repertoire, it is worth mentioning that the previous study demonstrated the rich catalog of secondary metabolites produced by *A. terreus* ATCC 20542 in addition to its main product, lovastatin (Boruta and Bizukojc 2016). Some of these molecules, e.g., (+)-geodin, asterric acid, and butyrolactone I, display interesting biological activities, e.g., act as enzyme inhibitors or antibiotics, what makes them the potential candidates for future drug discovery efforts (Kitagawa et al. 1994; Ohashi et al. 1992; Rinderknecht et al. 1947; Sato et al. 2005; Shinohara et al. 2000; Takatsuki et al. 1969). As the capability to produce a certain set of secondary metabolites is known to be strain-specific, studying the biosynthetic gene clusters in *A. terreus* ATCC 20542 still requires a fully sequenced genome. In our previous study, the dataset corresponding to *A. terreus* NIH2624 was chosen to be applied for bioinformatic analyses of fungal gene clusters solely due to the fact that the genome of our true experimental subject, namely *A. terreus* ATCC 20542, was still unavailable (Boruta and Bizukojc 2014).

Six out of seven complete genomes of *A. terreus* previously deposited into the NCBI database were released between the years 2016 and 2020, what reflects the recently observed rapid build-up of sequence data related to this microorganism. Generally, as the number of sequenced genomes of fungi representing a particular species increases, the door opens for comparative genomic analysis at the species level. It should be emphasized that the strains of *A. terreus* previously subjected to sequencing were isolated from diverse environments and represented various characteristics. Until now, there has been no comprehensive study addressing the genomic differences and similarities within the rapidly expanding group of sequenced *A. terreus* strains.

The goals of the present study were to provide a high-quality whole-genome assembly and annotation of *A. terreus* ATCC 20542 and to assess the diversity observed among the sequenced genomes of *A. terreus* strains.

## Materials and methods

### Cultivation conditions

*A. terreus* ATCC 20542 was purchased from the American Type Culture Collection (ATCC) and inoculated onto agar slants according to the recommendations specified by the ATCC. The spores obtained on the slants were then employed for the inoculation of liquid medium.

The submerged cultivations were carried out with the use of Certomat® BS-1 (B. Braun Biotech International, Melsungen, Germany) rotary shaker. The temperature was set at 28°C, the rotary speed was kept at 110 min^−1^. The fungal biomass proliferated in flat-bottom flasks (working volume 150 ml, total volume 500 ml).

The first stage of the cultivation process (i.e., the preculture) was initiated by transferring the spores to liquid medium (10^9^ fungal spores per liter of medium) with the use of a sterile pipette. After 24 h of cultivation, the second stage of the process was initiated by inoculating 150 ml of the production medium with 7 ml of the preculture. The process was continued for 48 h, and then, the biomass was separated from the liquid medium by filtration. The harvested biomass (fungal pellets) was washed thoroughly with PBS buffer (phosphate-buffered saline) and stored at −80°C.

The medium composition was as follows: lactose, 20 g l^−1^ (10 g l^−1^ in preculture); yeast extract, 4 g l^−1^ (8 g l^−1^ in preculture); KH_2_PO_4_, 1.51 g l^−1^; NaCl, 0.4 g l^−1^; MgSO_4_·7H_2_O, 0.51 g l^−1^; biotin, 0.04 mg l^−1^; and 1 ml l^−1^ of trace element solution of the following composition: ZnSO_4_·7H_2_O, 1 g l^−1^; Fe(NO_3_)_3_·9H_2_O, 2 g l^−1^; MnSO_4_, 50 mg l^−1^; Na_2_B_4_O_7_·10H_2_O, 100 mg l^−1^; Na_2_MoO_4_·2H_2_O, 50 mg l^−1^; and CuSO_4_·5H_2_O, 250 mg l^−1^. The sterilization was performed in an autoclave for 30 min at 121°C. The pH was set to 6.5 with the use of NaOH solution prior to sterilization.

### Isolation and sequencing of genomic DNA

Genomic DNA of *A. terreus* ATCC 20542 was isolated using the SDS/Phenol method as described previously (Wilson 1987; Nowak et al. 2019). DNA quality control was performed by measuring the absorbance at 260/230, template concentration was determined using Qubit fluorimeter (Thermo Fisher Scientific, Waltham, USA), and DNA integrity was analyzed by 0.8% agarose gel electrophoresis and by PFGE using Biorad CHEF-III instrument (BioRad, Hercules, USA).

Paired-end sequencing library was constructed using the NEB Ultra II FS Preparation Kit (New England Biolabs, Beverly, USA) according to the manufacturer’s instructions. Library was sequenced using an Illumina MiSeq platform (Illumina, San Diego, USA) with 2 × 300 paired-end reads. Sequence quality metrics were assessed using FASTQC (http://www.bioinformatics.babraham.ac.uk/projects/fastqc/; (Andrews 2010)).

The long reads were obtained using the MinION sequencer (Oxford Nanopore Technologies (ONT), Oxford, UK). Prior to long-read library preparation, genomic DNA was sheared into 20 kb fragments using Covaris g-Tube (Covaris, MA, USA) followed by size selection on Bluepippin instrument (Sage Science, Beverly, USA). DNA fragments above 10 kb were recovered using PAC30kb cassette. A total of 5 μg of recovered DNA was taken for 1D library construction using a SQK-LSK109 kit (Oxford Nanopore Technologies (ONT), Oxford, UK) and 0.5 μg of final library was loaded into a R9.4.1 flowcell (Oxford Nanopore Technologies (ONT), Oxford, UK) and sequenced on MinION sequencer (Oxford Nanopore Technologies (ONT), Oxford, UK).

### Genome assembling

Raw nanopore data was basecalled using Guppy (v. 3.0; Oxford Nanopore Technologies, Oxford, UK). After quality filtering using NanoFilt (De Coster et al. 2018) and residual adapter removal using Porechop (https://github.com/rrwick/Porechop), obtained dataset was quality checked using NanoPlot (De Coster et al. 2018). Long nanopore reads were assembled using Flye (v. 2.5; (Kolmogorov et al. 2019)). Flye-assembled contigs were further polished using Illumina sequencing reads and Oxford Nanopore ont-assembly-polish pipeline (https://github.com/nanoporetech/ont-assembly-polish).

### Annotation and analysis of *A. terreus* ATCC 20542 and the published *A. terreus* genomes

Genome assemblies of 8 *A. terreus* strains were downloaded from NCBI (access date: March 2020). Sequences of all analyzed *A. terreus* genomes were annotated using AUGUSTUS (v. 3.3.2; (Stanke and Waack 2003)) employing the supplied training annotation files for *A. terreus*. Ribosomal RNA (rRNA) genes were predicted in the *A. terreus* ATCC 20542 genome using barrnap (v. 0.9; Seemann T., https://github.com/tseemann/barrnap). Prediction of transfer RNA (tRNA) genes was done using the tRNAscan-SE (v. 2.0.5; (Chan and Lowe 2019)). Presence of signal peptide in the sequences of proteins was done using SignalP (v. 5.0; (Almagro Armenteros et al. 2019)). Transmembrane helices were predicted using Phobius server (access date: August 2020 (Käll et al. 2007)). Eukaryotic orthologous group (KOG) categories were assigned to the predicted *A. terreus* ATCC 20542 proteome by the WebMGA server (Wu et al. 2011). KEGG Automatic Annotation Server (KAAS; https://www.genome.jp/tools/kaas/) was used to perform KEGG orthology search employing the assignment method of ‘bi-directional best hit’ (BBH) against *Eurotiomycetes* species. The proteomes of the analyzed species were functionally characterized using software package InterProScan (v. 5.19; (Jones et al. 2014)) with the option to scan for Pfam collection of protein families. Carbohydrate-active enzymes (CAZyme; Lombard et al. 2014) were annotated using dbCAN2 meta server (Zhang et al. 2018) with the option to use HAMMER search against dbCAN HMM database, DIAMOND search against CAZy database, and Hotpep search against the PPR short peptide library. Candidate proteins found by at least two methods were kept for further analysis. Secondary metabolite clusters were predicted using antiSMASH for fungi (v. 5.1.1; (Medema et al. 2011; Blin et al. 2019)). Orthologs were searched between the proteomes using Proteinortho program (version 6.0.14; (Lechner et al. 2011)). ANI analysis performed using PYANI (v. 0.2.9) python program employing BLAST+ program (Camacho et al. 2009; Pritchard et al. 2016). The UPGM tree based on ANI–1 values was calculated using phangorn R package (Schliep 2011).

### Genome sequence and annotation data availability

The assembled genome, together with annotation, was deposited at the NCBI database under the BioProject: PRJNA622971.

## Results

### Sequence and general features of the genome of *A. terreus* ATCC 20542

**Fig. 1:**
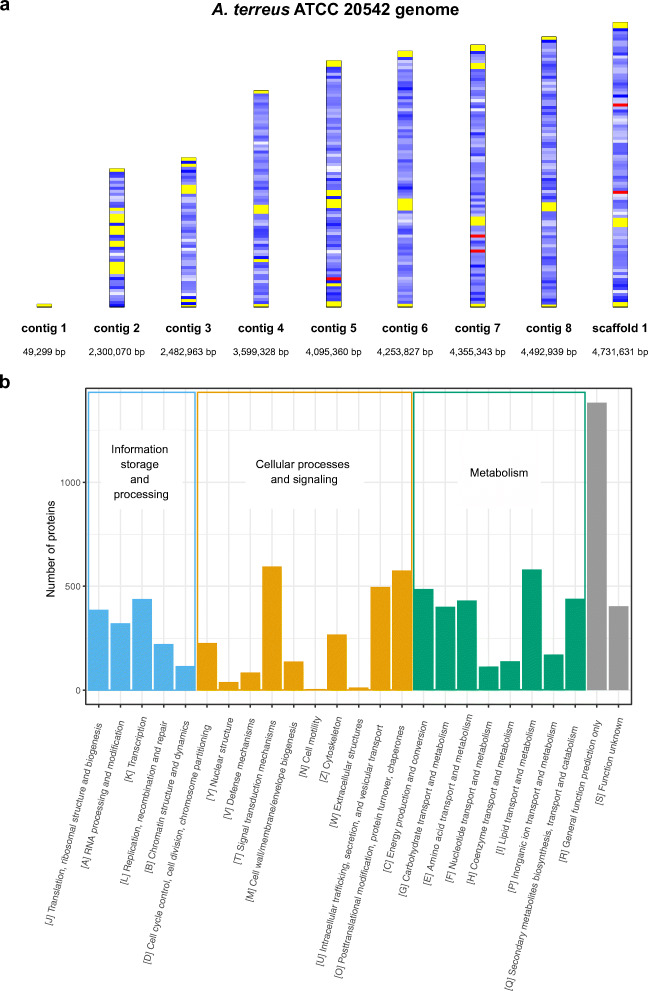
Characteristics of *A. terreus* ATCC 20542 genome. **a** Map of *A. terreus* ATCC 20542 genome composed of 8 contigs and one scaffold color-coded according to GC content (red is more than 57%, yellow less than 50%, and the blued shades represent a linear gradient between the both numbers). **b** KOG categories in *A. terreus* ATCC 20542 proteome annotated by the WebMGA web server. Proteins assigned to more than one category were counted in each of them

The sequencing and assembling of *A. terreus* ATCC 20542 genome produced 8 contigs and 1 scaffold of the total size of 30,360,760 bp and the GC content of 52.2% (Table 1; Fig. 1a). The estimated number of protein-coding genes in the genome was equal to 10,505. Forty-five rRNA genes were predicted. Out of 196 predicted tRNA genes, 18 were classified as pseudogenes which may not be functional. The signal peptide was detected in the sequence of 1025 proteins, whereas 2502 were predicted to contain transmembrane regions. Based on InterProscan search, Pfam domains were assigned to 8223 proteins (78% of the proteome). A total of 6533 of proteins were assigned to KOG categories representative of four main function types: information storage and processing, cellular processes and signaling, metabolism, and poorly characterized proteins (Fig. 1b).

**Table 1:** Assembly statistics and general features of *A. terreus* ATCC 20542 genome

Total number of reads-Illumina	17,481,351
Total number of reads–ONT	392,432
Genome size (bp)	30,360,760
Number of contigs/scaffolds	9
Scaffold N50 (bp)	4,253,827
Scaffold L50	3
% GC	52.2
Genome coverage-Illumina	163×
Genome coverage-ONT	140×
Number of protein coding genes	10,505
rRNA genes	45
tRNA genes	196
Proteins with signal peptide	1025
Proteins with transmembrane helices	2502
Proteins with predicted Pfam domain	8223
Proteins assigned to KOG	6,533
Predicted CAZy proteins	579

### Phylogenetic analysis and comparative genomics of *A. terreus* strains

The genomes of other seven *A. terreus* strains were downloaded from the NCBI database (Table 2). The average genome size of an *A. terreus* strain is 29.6 Mb and the average GC content is 52%. On average, the *A. terreus* genome encodes 10,421 genes (excluding the *A. terreus* T3 Kankrej genome due to high assembly fragmentation). Only the genome assembly of *A. terreus* NIH 2624 includes mitochondrial DNA sequence (1 scaffold of 32,827 bp with no predicted genes).

**Table 2:** Characteristics of complete *A. terreus* genomes deposited at the NCBI database. Genes were predicted by Augustus

Strain	GenBank assembly accession	Total genome size (bp)	Number of contigs/scaffolds	N50 (bp)	%GC	Number of predicted protein-coding genes
NIH 2624	GCA_000149615.1	29,364,022	27	1,912,493	52.6	10,413
45A	GCA_001630395.1	29,325,441	26	1,912,382	51.1	10,219
w25	GCA_002749855.1	29,423,947	536	326,294	52.7	10,436
T3 Kankrej	GCA_002930435.1	27,963,153	13,340	3497	52.1	13,346
TN-484	GCA_009014675.1	29,615,077	1211	673,704	52.4	10,347
M6925	GCA_009834425.1	31,839,144	35	4,133,337	52.2	10,798
IFO 6365	GCA_009932835.1	28,667,710	23	2,163,102	53.0	10,227

**Fig. 2:**
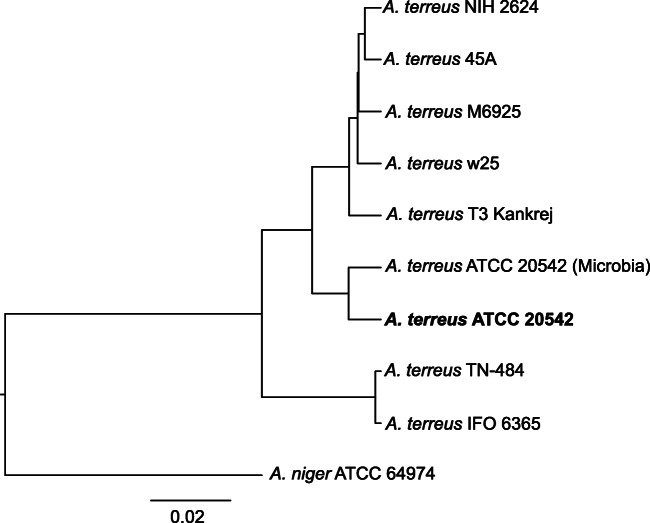
Phylogenetic tree of *A. terreus* strains based on ANI analysis performed using PYANI (0.2.9) python program employing BLAST+ program (Camacho et al. 2009; Pritchard et al. 2016). The UPGM tree based on ANI–1 values was calculated using phangorn R. package (Schliep 2011). The *A. terreus* ATCC 64974 was used as an outgroup. The tree was drawn in FigTree program (v. 1.4.4). The scale bar represents the sequence divergence

According to the results of the phylogenetic analysis based on the ANI values, three main clades can be noticed (Fig. 2), i.e., one which groups genomes of the *A. terreus* NIH 2624, *A. terreus* 45A, *A. terreus* M6925, *A. terreus* w25, and *A. terreus* T3 Kankrej strains; the second clade with two *A. terreus* ATCC 20542 genomes; the third and the most separated from the first two, clade grouping the genomes of IFO 6365 and TN-484 strains.

### Primary carbon metabolism and plant biomass degradation

In order to gain insight into the conservation of the primary metabolism pathways in *A. terreus* strains, first their potential to utilize carbohydrates was compared by investigating the presence of orthologs of respective catabolic enzymes based on sequence similarity with proteins of *Aspergillus niger* ATCC 1015 (Table S1 of Online Resource 1). The investigated carbon sources included cellobiose, D-fructose, D-galactose, D-glucose, lactose, D-mannose, maltose, sucrose, and D-xylose. A similar analysis was conducted with regard to the genes involved in the production of organic acids, such as citric, fumaric, malic, succinic, and gluconic acid (Table S2 of Online Resource 1). In general, orthologs of all the investigated enzymes were found in every *A. terreus* genome and their copy number agreed, apart from only a few cases such as, e.g., glucose oxidase and D-xylulose reductase.

Next, to evaluate the plant biomass-degrading ability across *A. terreus* strains, the genes from CAZy (Carbohydrate-Active enZYmes) families were predicted (Table 3). The total number of CAZy genes predicted in a single genome varied from 450 to 483 depending on the strain, with the highest number found in the genome of *A. terreus* M6925. The differences in the numbers of genes involved in the degradation of cellulose, xylan, galactomannan, and xyloglucan did not exceed 1 among the tested genomes, whereas the abundance of sequences associated with pectin, inulin, and starch turned out to be somewhat more varied (Table 3). The analysis revealed that the genome of *A. terreus* M6925 was particularly enriched in inulinase-encoding genes. It was also noticed that the genomes of the two *A. terreus* strains of the second phylogenetic clade, i.e., *A. terreus* IFO 6365 and TN-484, contained the lowest number of genes required for the degradation of cellulose, pectin, and starch.

**Table 3:** Number of carbohydrate active enzymes predicted in *A. terreus* genomes. Assignment of the CAZy families to polysaccharides was performed according to de Vries et al. (2017)

	Cellulose	Xylan	Galactomannan	Xyloglucan	Pectin	Starch	Inulin	Total
CAZy families/Strain	GH5_4, GH5_5, GH5_22, GH6, GH7, GH45, AA9	GH10, GH11, GH62, GH67, GH115, CE1, CE15	GH5_7, GH26, GH27, GH36, GH134	GH12, GH29, GH74, GH95	GH28, GH53, GH78, GH88, GH93, GH105, PL1, PL3, PL4, PL9, PL11, PL22, CE8, CE12	GH13_1, GH13_5, GH13_40, GH15, GH31, GH133	GH32	All
ATCC 20542	23	18	11	9	36	23	6	466
NIH 2624	23	18	12	9	35	22	6	467
45A	23	18	11	9	34	22	6	463
w25	23	18	11	10	37	22	6	474
IFO 6365	22	18	11	9	33	21	6	450
TN-484	22	18	11	9	33	21	6	452
M6925	23	17	11	10	37	23	11	483

Even though the set of genes associated with primary metabolic pathways in *A. terreus* ATCC 20542 at first glance does not seem to be exceptional compared with other representatives of the species, a small set of unique sequences having no counterparts in other *A. terreus* strains has been found throughout the analysis (Table S3 of Online Resource 1). The example of such a sequence is the gene of a putative hexokinase (gene ID: HFD88_003314), for which the best BLASTP hits in the non-redundant protein database were the industrial progenitor strain *Penicillium chrysogenum* P2niaD18 and *Penicillium rubens* 43M1 (80% identity in both cases), whereas no *A. terreus* counterparts could be found. Another example is the gene of a putative catalase (gene ID: HFD88_003313), which closely resembles the sequences found in other *Aspergillus* species (e.g., 89% identity with *Aspergillus ruber CBS* 135680 gene) but was not detected in any available genome of *A. terreus*. Interestingly, the genome of *A. terreus* ATCC 20542 carries a putative amidase gene that is generally missing in *Aspergilli* but exhibits more than 55% identity with the sequences of more evolutionarily distant fungal genera, namely *Exophiala*, *Fonsecaea*, and *Cladophialophora*.

### Secondary metabolism

**Fig. 3:**
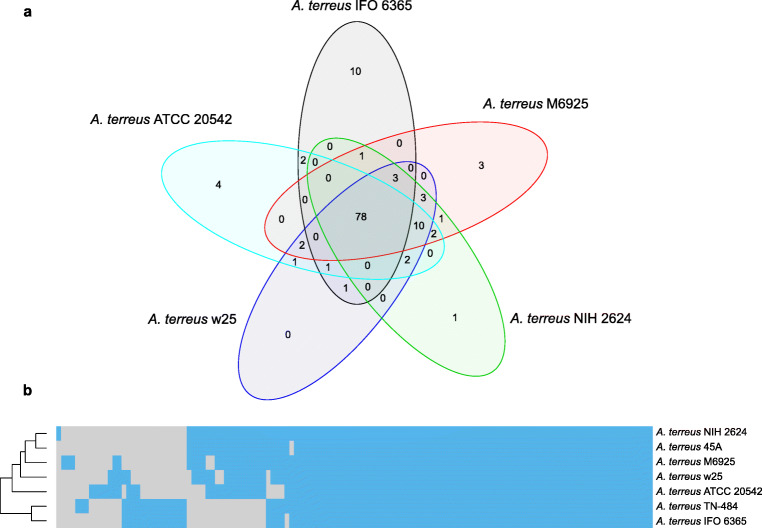
Comparison of the predicted secondary metabolism potential in *A. terreus* strains. **a** Venn diagram of SM proteins from 5 representative genomes of each clade. The representative genomes were selected based on the phylogenetic analysis and genome quality. **b** Clustering of the *A. terreus* strains based on presence (blue tiles)/absence (gray tiles) of orthologous core biosynthetic proteins involved in secondary metabolism predicted by antiSMASH in every genome. Dendrogram was generated based on hierarchical clustering analysis. *Y*-axis: strain clustering; *x*-axis: protein clustering (dendrogram and orthologous groups not shown). *A. terreus* T3 Kankrej was not included in the analysis due to unsatisfactory quality of its genome (high sequence fragmentation)

On the basis of the prediction, *A. terreus* ATCC 20542 genome encodes 74 secondary metabolite biosynthesis gene clusters (also referred to as “regions” according to the nomenclature used in antiSMASH 5 genome mining pipeline). Each of these regions is represented by at least one core biosynthetic gene representing polyketide synthase (PKS), non-ribosomal peptide synthetase (NRPS), terpene cyclase (TC), dimethylallyl tryptophan synthase (DMATS), or hybrids of various biosynthetic groups. These clusters are encoded on every *A. terreus* ATCC 20542 contig and one scaffold, except for the smallest, contig 1 (the Online Resource 1: Fig. S1). When other *A. terreus* genomes are considered, it can be noticed that the total number of predicted core biosynthetic genes involved in secondary metabolite (SM) production varied from 94 in *A. terreus* IFO 6365 to 99 in *A. terreus* M6925 (Table 4). Analysis of conservation of the core biosynthetic genes has shown that 78 of them are present in all of the analyzed *A. terreus* genomes (Fig. 3a). The genome of the main subject of this study, namely *A. terreus* ATCC 20542, is predicted to encode four unique core genes. The highest number (10) of unique sequences is encoded by the *A. terreus* IFO 6365 and TN-484 genomes (Fig. 3 a and b). Moreover, these two genomes do not encode a large group of over 15 SM core genes which are conserved in other *A. terreus* genomes (Fig. 3b).

**Table 4:** Core biosynthetic genes of *A. terreus* strains predicted by antiSMASH based on Augustus annotations

Type/strain	Type I iterative PKS	NRPS	NRPS-like	PKS/NRPS-like	Hybrid	TC (terpene)	DMATS (indole)	Total number core biosynthetic genes
ATCC 20542	25	24	22	5	2	8	10	96
NIH 2624	27	19	26	3	2	10	10	97
45A	26	19	25	4	2	9	10	95
w25	28	19	25	3	2	10	10	97
IFO 6365	27	15	29	2	5	8	8	94
TN-484	28	16	29	2	5	8	10	98
M6925	26	20	29	3	2	9	10	99

*PKS* polyketide synthase, *NRPS* non-ribosomal peptide synthetase, *TC* terpene cyclase, *DMATS* dimethylallyl tryptophan synthase

**Fig. 4:**
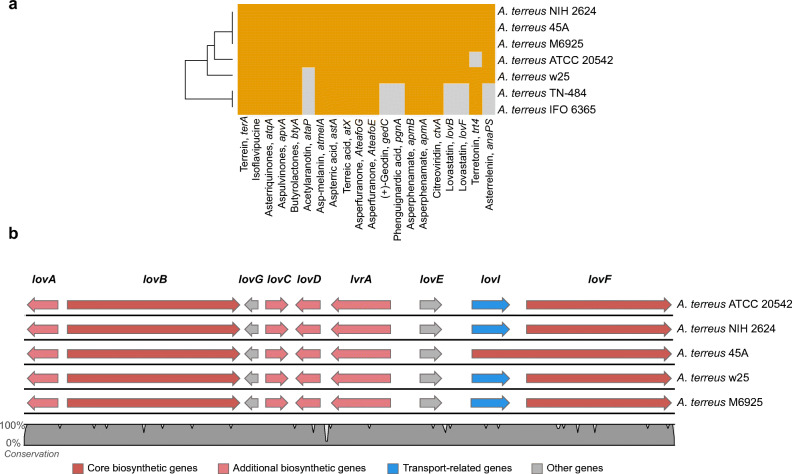
Comparison of the characterized secondary metabolism gene clusters in *A. terreus* strains. **a** Clustering of the *A. terreus* strains based on presence (blue tiles)/absence (gray tiles) of orthologs of *A. terreus* NIH 2624 core biosynthetic proteins involved in secondary metabolism, which were experimentally linked to their metabolic products in previous studies (as reviewed by Romsdahl and Wang 2019). Dendrogram was generated based on hierarchical clustering analysis. *Y*-axis: strain clustering; *x*-axis: protein clustering (dendrogram not shown). *A. terreus* T3 Kankrej was not included in the analysis due to unsatisfactory quality of its genome (high sequence fragmentation). **b** Comparison of lovastatin biosynthesis gene cluster conserved in five *A. terreus* genomes (gene names according to Dietrich and Vederas 2014)

Analysis of conservation pattern of core SM synthases, which were experimentally linked to downstream products (as reviewed by Romsdahl and Wang 2019), has highlighted some distinctive features among the *A. terreus* genomes (Fig. 4a). For example, *A. terreus* ATCC 20542 genome does not encode the terretonin gene cluster (the core gene and the sequences of accessory proteins are all missing). The *A. terreus* IFO 6365 and TN-484 genomes, which are also the most phylogenetically distant from the other *A. terreus* genomes, do not encode acetylaranotin (*ataP*), (+)-geodin (*gedC*), phenguignardic acid (*pgnA*), lovastatin (*lovB* and *lovF*), nor the asterrelenin, epi-aszonalenin A (*anaPS*) genes. The core genes of acetylaranotin production turned out to be the least conserved among the sequences considered in the present analysis, as they are missing in three out of seven investigated *A. terreus* strains (Fig. 4a). The biosynthetic gene cluster of lovastatin, the key secondary metabolite produced by *A. terreus*, was demonstrated here to be well-conserved among the five of the sequenced genomes of this species (Fig. 4b). Still, several single-nucleotide polymorphisms (SNPs) could be noticed along the cluster (Fig. 4b). The genes *lovI* and *lovF* turned out to be merged into a single sequence in *A. terreus* 45A genome, but this may be due to incorrect in silico predictions and still requires further experimental verification.

Since the genome of *A. terreus* ATCC 20542 is the main focus of this study, a putative biosynthetic gene cluster that, as a whole, is unique to the newly sequenced strain was further analyzed in detail. This cluster encompasses the sequences spanning from HFD88_004290 to HFD88_004297 (contig 4th: 885,353..929,704) with HFD88_004294 (annotated as a PKS sequence) as the core biosynthetic gene. The amino acid sequence of the putative PKS encoded by HFD88_004294 has counterparts neither in the sequenced *A. terreus* strains, nor in any genome previously deposited into the NCBI. In this case, the closest BLASTP hit against the non-redundant protein database is the sequence found in *Aspergillus brasiliensis* (66% identity at 68% query coverage). For comparison, if the sequence of lovastatin nonaketide synthase (LovB) originating from *A. terreus* NIH2624 is used as a query in BLASTP search using the same database, the percentage identity with the LovB counterpart found previously in *A. terreus* ATCC 20542 (Hendrickson et al. 1999) reaches as much as 98% at 100% query coverage. As far as the remaining regions of the above-mentioned unique cluster are concerned, three sequences in this region (HFD88_004290, HFD88_004291 and HFD88_004293) display close resemblance (more than 90% identity) with the proteins encoded in the genome of *Aspergillus caelatus*, whereas for HFD88_004295 and HFD88_004297, the best BLASTP hits were recorded in the genome of *Coccidioides posadasii*. For HFD88_004296, the most similar protein was detected in *A. brasiliensis*, similarly as for the core biosynthetic protein. Finally, there is only one sequence in this region that has its well-conserved counterpart in a strain representing the *A. terreus* species, namely HFD88_004292, which is 98% identical with the corresponding orthologous protein in *A. terreus* NIH 2624.

## Discussion

Until now, only a small portion (138,520 bp) of the *A. terreus* ATCC 20542 genome was made available to the public (provided by Microbia company). To fill this information gap and to provide the scientific community with the genome of this industrially important strain, we sequenced it using a hybrid approach consisting of long (Oxford Nanopore Technologies (ONT), Oxford, UK) and short read sequences (Illumina, San Diego, USA). The resulting assembly was of 30.4 Mb and consisted of 8 contigs and 1 scaffold. Comparison with other *A. terreus* assemblies has shown that, in terms of the continuity-related quality of the genome, there are no previously released *A. terreus* assemblies that could be regarded as superior to the one presented in the current work (Table 2). Moreover, the analysis revealed that the size and general features of the *A. terreus* ATCC 20542 genome are comparable with other sequenced *A. terreus* genomes.

Availability of the whole genome sequence of the *A. terreus* strains opened the possibility to precisely measure the level of similarity displayed within this species. Phylogenetic analysis of the eight *A. terreus* strains highlighted the considerable genomic distance between them. The phylogenetic tree built on the ANI values consisted of three main clades, where the most occupied clade was represented by the *A. terreus* NIH 2624 strain and 4 other strains. On this tree, *A. terreus* ATCC 20542 formed a separate clade, and the most distant clade from the two clades was represented by the *A. terreus* IFO 6365 and *A. terreus* TN-484 strains. These findings suggested that the genetic differences between the *A. terreus* strains may be greater than expected and that they may directly influence the biosynthetic capabilities of these strains, which make them worth exploring.

First, we focused on the primary metabolism. According to the annotation presented in the current study, *A. terreus* ATCC 20542 is equipped with genes of catabolic enzymes allowing for the utilization of a broad set of carbon sources such as lactose and D-galactose (Table S1 of Online Resource 1). Among these carbon sources, it should be pointed out that lactose is the substrate of exceptional importance with regard to *A. terreus* ATCC 20542 cultivations. This slowly metabolized disaccharide was considered by many authors to be the recommended carbon source in the context of elevating lovastatin titers (Mulder et al. 2015). The catabolism of lactose encompasses several enzymatic steps, starting with its hydrolysis catalyzed by β-galactosidase to yield D-glucose (which then enters glycolysis) and D-galactose. In most eukaryotic organisms, D-galactose undergoes conversion into D-glucose-1-phosphate by the catalytic action of galactokinase, galactose-1-phosphate uridylyltransferase, and UDP-galactose 4-epimerase (Seiboth et al. 2007). The genes encoding the enzymes responsible for this metabolic route, known as the Leloir pathway, have all been found in the annotated genome of *A. terreus* ATCC 20542 (Table S1 of Online Resource 1). It is worth mentioning that these three genes were detected only in single copies, what agrees well with the findings of de Vries et al. (2007), who previously examined 19 genomes of various species representing the *Aspergillus* genus. In the mentioned study, the sequences of galactokinase and UDP-galactose 4-epimerase were found in a single copy in all the investigated assemblies, whereas the gene of galactose-1-phosphate uridylyltransferase turned out to be present in two copies in *Aspergillus glaucus* and in a single copy in the remaining 18 *Aspergilli*.

In a species-wide perspective, the comparative analysis revealed only a modest degree of diversity with respect to the presence and the number of genes involved in carbon source utilization in *A. terreus* strains (Table S1 of Online Resource 1). In some cases, the number of orthologous sequences associated with a given enzymatic function was not identical in all the investigated fungi (e.g., the number of invertase genes varied from 1 to 2 depending on the microorganism), but for most sequences, encoding the sugar catabolic enzymes, no diversity was recorded in this respect (Table S1 of Online Resource 1). Similar observations were made with regard to the genes involved in the production of organic acids, where even slight differences in gene copy numbers were very rare among the tested strains (Table S2 of Online Resource 1). Moreover, evaluation of the plant biomass-degrading ability across *A. terreus* strains, based on presence of the genes from CAZy families, has shown some modest degree of diversity among them. Especially the genome of *A. terreus* M6925 was found to harbor the highest number of CAZy genes among all of the investigated strains. Moreover, the two phylogenetically most distant strains, namely *A. terreus* IFO 6365 and TN-484, were predicted to have slightly poorer enzyme repertoire for the degradation of cellulose, pectin, and starch, in comparison with other *A. terreus* species. In general, these results may be seen as a correlation between the phylogenetic relationship between the strains and the similarity of genetic inventories related to plant biomass degradation.

All in all, as far as the catabolic enzyme-encoding genes associated with the metabolism of sugars, organic acids, and plant biomass components are concerned, this functionally related part of the *A. terreus* genome is rather well-conserved at the species level and the genetic diversity among the strains of *A. terreus* can be regarded as relatively low. However, some subtle, yet distinguishable features for each strain can always be found. For example, in the newly sequenced genome of *A. terreus* ATCC 20542, we found several unique genes coding for, e.g., hexokinase, catalase, or amidase which had no counterparts among *A. terreus* strains bet shared similarity with more evolutionarily distant fungal genera (Table S3 of Online Resource 1). One cannot exclude that these unique genes played a role in the survival of *A. terreus* ATCC 20542 in its natural environment and contributed to its overall fitness and metabolic capabilities.

The next metabolic pathways which were analyzed were those related to secondary metabolism. Compared with other strains, the newly sequenced genome of *A. terreus* ATCC 20542 turned out to be particularly rich in NRPS genes, what might reflect the potential of this microorganisms to produce a relatively wide spectrum of non-ribosomal peptides (Table 4). According to the antiSMASH analysis performed in the present work, all of the investigated *A. terreus* strains are equipped with 10 DMATS genes except *A. terreus* IFO6365, which has only eight genes of this kind. As recently mentioned by Takahashi at el. (2020), *A. terreus* IFO6365 is “one of the highest-yielding itaconic acid-producing wild-type strains”. It is interesting that the mutant derived from this strain, namely *A. terreus* TN-484, is more enriched with the core biosynthetic genes associated with SM production than its parental strain, having more genes encoding PKS, NRPS, and TC enzymes (Table 4). In fact, the genomes of *A. terreus* TN-484 and *A. terreus* w25 harbor the largest number of PKS genes among all the investigated datasets (Table 4). Having a broader set of biosynthetic genes could be seen as an unnecessary burden for a mutant strain tailored specifically towards the efficient production of itaconic acid, even if these extra genes remained silent under typical cultivation conditions. When conservation pattern of the core biosynthetic genes was analyzed, it was revealed that it followed the phylogenetic relationship among the *A. terreus* strains (Fig. 3b). Considering the number of core genes conserved only among a limited number of strains or present solely in a single strain, one may expect a strain-specific catalog of secondary metabolites exhibited by the representatives of this fungal species. Particularly, the *A. terreus* IFO 6365 and TN-484 genomes displayed here the most distinguishable features. These genomes were not only found to harbor the highest number of unique sequences but also the largest group of core SM genes was not conserved in them. Among the absent genes encoding some of the experimentally verified SM clusters, such as those of lovastatin and (+)-geodin (Fig. 4a). Since lovastatin and (+)-geodin are the secondary metabolites produced in relatively large quantities in *A. terreus* cultures (Bizukojc and Ledakowicz 2007; Hasan et al. 2019), the lack of genes responsible for the biosynthesis of these two molecules in itaconic acid producers *A. terreus* IFO 6365 and TN-484 can be viewed as a relief of the unnecessary biosynthetic load in favor of maximizing itaconic acid yields. Several features related to SM synthesis were also discovered in the *A. terreus* ATCC 20542 genome. For example, it lacks terretonin gene cluster but harbors four unique core genes. By further investigating of these genes, a completely unique gene cluster was exposed, which as a whole is present only in *A. terreus* ATCC 20542 and no other *A. terreus* strains. The core biosynthetic gene of this cluster, a putative PKS sequence, was found to be particularly unique since it has no counterparts in any of the genomes deposited at the NCBI database. This cluster can be regarded as interesting targets of future experimental efforts towards uncovering novel secondary metabolites of potential industrial and pharmaceutical interest.

The predicted absence of the core genes from the terretonin and (+)-geodin pathway in *A. terreus* ATCC 20542 and both, *A. terreus* IFO 6365 and TN-484, respectively, may be important in the context of invasive fungal infections, since these metabolites have been identified as pathogenicity factors in *A. terreus* (reviewed in Bengyella et al. 2017 and Shankar et al. 2018). It is possible that the absence of these genes as well as those of other predicted SM gene clusters may influence pathogenic potential of these strains. Indeed, *A. terreus* ATCC 20542 is classified as unlikely to cause disease (Biosafety level 1, BSL1). Further research, however, is needed to verify and to relate the absence of these metabolites with pathogenicity of these strains. Nevertheless, the SM gene clusters predicted in this study may constitute a valuable resource for searching of new pathogenic factors which would distinguish pathogenic strains from non-pathogenic ones, since the key virulent proteins which were identified in a proteomic study of *A. terreus* (Thakur and Shankar 2017) are all present in the genomes of *A. terreus* strains (Table S4 of Online Resource 1). It is also possible that sequence polymorphism at the regulatory regions modulating expression of these genes may exist. In order to identify it and to investigate its influence, more studies comparing expression level of these genes among the *A. terreus* strains are needed.

As discussed above, the genome of *A. terreus* ATCC 20542 harbors several genes that can be regarded as unique among the sequenced genomes of *A. terreus*. Further investigation of the biological events associated with these sequences, e.g., the analysis of expression, metabolic profiles recorded for the deletion mutants or gene-by-gene elucidation of the biosynthetic gene clusters, would surely provide insights into the underlying molecular mechanisms associated with the phenotypic diversity at the species level. Furthermore, the complete genome of a model lovastatin-producing strain will open the door for the system-scale studies on lovastatin biosynthesis, possibly leading to improved, highly efficient fungal cell factories obtained via metabolic engineering. An interesting study would be to comprehensively compare the wild-type parental strain with its progeny of industrial mutants obtained mostly by random mutagenesis to gain insights into cellular changes that ultimately lead to increased lovastatin titers, productivities, and yields. Unfortunately, since the complete genomes of lovastatin-producing industrial mutants of *A. terreus* have not been released so far, the analysis of this kind is not feasible at the moment. Addressing the genomic diversity of *A. terreus*, there are considerable differences among the genetic inventories associated with secondary metabolic pathways in the strains belonging to this species. Moreover, there are certain links between the known characteristics of the investigated fungi and their genomic features, e.g. the lack of lovastatin and (+)-geodin clusters in the strains used for the production of itaconic acid. As the predictions regarding the biosynthetic gene clusters indicate, understanding the species-level biosynthetic diversity in *A. terreus* can be expected to facilitate the process of strain identification based on metabolic profiles.

To conclude, the present study provides the complete genome sequence of lovastatin-producing wild-type strain *A. terreus* ATCC 20542. In terms of continuity, this is the dataset of highest quality among the released *A. terreus* genomes. The assembled and annotated genome of this model microorganism will serve as a future reference for studying the biosynthesis of lovastatin and other fungal secondary metabolites. The comparative study conducted here shows that the diversity among *A. terreus* strains is more evident in the genomic areas associated with secondary metabolism than in the segments encoding the genes related to primary metabolic pathways; however, the differences in the number of highly conserved catabolic genes are also clearly visible. Finally, the newly sequenced genome of *A. terreus* ATCC 20542 harbors several unique sequences that are interesting subjects for future research on fungal metabolism, especially in the comparative context.

## Supplementary information

ESM 1 (PDF 324 kb)

## Data Availability

The sequence of *A. terreus* ATCC 20542 genome is accessible through NCBI database under the BioProject: PRJNA622971.

## References

[CR1] Alberts AW, Chen J, Kuron G, Hunt V, Huff J, Hoffman C, Rothrock J, Lopez M, Joshua H, Harris E, Patchett A, Monaghan R, Currie S, Stapley E, Albers-Schonberg G, Hensens O, Hirshfield J, Hoogsteen K, Liesch J, Springer J (1980). Mevinolin: a highly potent competitive inhibitor of hydroxymethylglutaryl-coenzyme A reductase and a cholesterol-lowering agent. Proc Natl Acad Sci U S A.

[CR2] Almagro Armenteros JJ, Tsirigos KD, Sønderby CK, Petersen TN, Winther O, Brunak S, von Heijne G, Nielsen H (2019). SignalP 5.0 improves signal peptide predictions using deep neural networks. Nat Biotechnol.

[CR3] Andrews S (2010) FastQC: a quality control tool for high throughput sequence data. Available online at: http://www.bioinformatics.babraham.ac.uk/projects/fastqc

[CR4] Askenazi M, Driggers EM, Holtzman DA, Norman TC, Iverson S, Zimmer DP, Boers M-E, Blomquist PR, Martinez EJ, Monreal AW, Feibelman TP, Mayorga ME, Maxon ME, Sykes K, Tobin JV, Cordero E, Salama SR, Trueheart J, Royer JC, Madden KT (2003). Integrating transcriptional and metabolite profiles to direct the engineering of lovastatin-producing fungal strains. Nat Biotechnol.

[CR5] Barrios-González J, Miranda RU (2010). Biotechnological production and applications of statins. Appl Microbiol Biotechnol.

[CR6] Bengyella L, Yekwa EL, Subhani MN, Tambo E, Nawaz K, Hetsa BA, Iftikhar S, Walkhom SD, Roy P (2017). Invasive *Aspergillus terreus* morphological transitions and immunoadaptations mediating antifungal resistance. Infect Drug Resist.

[CR7] Bizukojc M, Ledakowicz S (2007). Simultaneous biosynthesis of (+)-geodin by a lovastatin-producing fungus *Aspergillus terreus*. J Biotechnol.

[CR8] Blin K, Shaw S, Steinke K, Villebro R, Ziemert N, Lee SY, Medema MH, Weber T (2019). AntiSMASH 5.0: updates to the secondary metabolite genome mining pipeline. Nucleic Acids Res.

[CR9] Boruta T, Bizukojc M (2014). Culture-based and sequence-based insights into biosynthesis of secondary metabolites by *Aspergillus terreus* ATCC 20542. J Biotechnol.

[CR10] Boruta T, Bizukojc M (2016). Induction of secondary metabolism of *Aspergillus terreus* ATCC 20542 in the batch bioreactor cultures. Appl Microbiol Biotechnol.

[CR11] Buckland B, Gbewonyo K, Hallada T, Kaplan L, Masurekar P, Demain AL, Somkuti GA, Hunter-Cevera JC, Rossmore HW (1989). Production of lovastatin, an inhibitor of cholesterol accumulation in humans. Novel Microbial Products for Medicine and Agriculture.

[CR12] Camacho C, Coulouris G, Avagyan V, Ma N, Papadopoulos J, Bealer K, Madden TL (2009). BLAST+: architecture and applications. BMC Bioinf.

[CR13] Chan PP, Lowe TM (2019). tRNAscan-SE: Searching for tRNA genes in genomic sequences. Methods Mol Biol.

[CR14] De Coster W, D’Hert S, Schultz DT, Cruts M, Van Broeckhoven C (2018). NanoPack: visualizing and processing long-read sequencing data. Bioinformatics.

[CR15] de Vries RP, Riley R, Wiebenga A, Aguilar-Osorio G, Amillis S, Uchima CA, Anderluh G, Asadollahi M, Askin M, Barry K, Battaglia E, Bayram Ö, Benocci T, Braus-Stromeyer SA, Caldana C, Cánovas D, Cerqueira GC, Chen F, Chen W, Choi C, Clum A, dos Santos RAC, de Lima Damásio AR, Diallinas G, Emri T, Fekete E, Flipphi M, Freyberg S, Gallo A, Gournas C, Habgood R, Hainaut M, Harispe ML, Henrissat B, Hildén KS, Hope R, Hossain A, Karabika E, Karaffa L, Karányi Z, Kraševec N, Kuo A, Kusch H, LaButti K, Lagendijk EL, Lapidus A, Levasseur A, Lindquist E, Lipzen A, Logrieco AF, MacCabe A, Mäkelä MR, Malavazi I, Melin P, Meyer V, Mielnichuk N, Miskei M, Molnár ÁP, Mulé G, Ngan CY, Orejas M, Orosz E, Ouedraogo JP, Overkamp KM, Park HS, Perrone G, Piumi F, Punt PJ, Ram AFJ, Ramón A, Rauscher S, Record E, Riaño-Pachón DM, Robert V, Röhrig J, Ruller R, Salamov A, Salih NS, Samson RA, Sándor E, Sanguinetti M, Schütze T, Sepčić K, Shelest E, Sherlock G, Sophianopoulou V, Squina FM, Sun H, Susca A, Todd RB, Tsang A, Unkles SE, van de Wiele N, van Rossen-Uffink D, de Castro Oliveira JV, Vesth TC, Visser J, Yu JH, Zhou M, Andersen MR, Archer DB, Baker SE, Benoit I, Brakhage AA, Braus GH, Fischer R, Frisvad JC, Goldman GH, Houbraken J, Oakley B, Pócsi I, Scazzocchio C, Seiboth B, vanKuyk PA, Wortman J, Dyer PS, Grigoriev IV (2017). Comparative genomics reveals high biological diversity and specific adaptations in the industrially and medically important fungal genus *Aspergillus*. Genome Biol.

[CR16] Dietrich D, Vederas JC, Martín JF, García-Estrada C, Zeilinger S (2014). Lovastatin, compactin, and related anticholesterolemic agents. Biosynthesis and molecular genetics of fungal secondary metabolites. Fungal biology.

[CR17] Endo A (2010). A historical perspective on the discovery of statins. Proc Jpn Acad Ser B.

[CR18] Ghanem NB, Yusef HH, Mahrouse HK (2000). Production of *Aspergillus terreus* xylanase in solid-state cultures: application of the Plackett-Burman experimental design to evaluate nutritional requirement. Bioresour Technol.

[CR19] Hasan H, Abd Rahim MH, Campbell L, Carter D, Abbas A, Montoya A (2019). Improved lovastatin production by inhibiting (+)-geodin biosynthesis in *Aspergillus terreus*. New Biotechnol.

[CR20] Hendrickson L, Davis CR, Roach C, Nguyen DK, Aldrich T, McAda PC, Reeves CD (1999). Lovastatin biosynthesis in *Aspergillus terreus*: characterization of blocked mutants, enzyme activities and a multifunctional polyketide synthase gene. Chem Biol.

[CR21] Jones P, Binns D, Chang H-Y, Fraser M, Li W, McAnulla C, McWilliam H, Maslen J, Mitchell A, Nuka G (2014). InterProScan 5: genome-scale protein function classification. Bioinformatics.

[CR22] Käll L, Krogh A, Sonnhammer ELL (2007) Advantages of combined transmembrane topology and signal peptide prediction-the Phobius web server. Nucleic Acids Res (Web Server issue):W429–W432 10.1093/nar/gkm256 PMC1933244 17483518

[CR23] Kanamasa S, Minami T, Okabe M, Park EY, Fujimoto T, Takahashi A, Murase M, Fukuyoshi S, Oda A, Satou K, Takahashi H (2019). Draft genome sequence of *Aspergillus terreus* high-itaconic-acid-productivity mutant TN-484. Microbiol Resour Announc.

[CR24] Kennedy J, Auclair K, Kendrew SG, Park C, Vederas JC, Hutchinson CR (1999). Modulation of polyketide synthase activity by accessory proteins during lovastatin biosynthesis. Science.

[CR25] Kitagawa M, Higashi H, Takahashi IS, Okabe T, Ogino H, Taya Y, Hishimura S, Okuyama A (1994). A cyclin-dependent kinase inhibitor, butyrolactone I, inhibits phosphorylation of RB protein and cell cycle progression. Oncogene.

[CR26] Klement T, Buchs J (2013). Itaconic acid–a biotechnological process in change. Bioresour Technol.

[CR27] Kolmogorov M, Yuan J, Lin Y, Pevzner PA (2019). Assembly of long, error-prone reads using repeat graphs. Nat Biotechnol.

[CR28] Krull S, Hevekerl A, Kuenz A, Prüße U (2017). Process development of itaconic acid production by a natural wild type strain of *Aspergillus terreus* to reach industrially relevant final titers. Appl Microbiol Biotechnol.

[CR29] Lass-Flörl C (2018) Treatment of infections due to *Aspergillus terreus* species complex. J Fungi (Basel) 4(83) 10.3390/jof4030083 PMC6162764 29987241

[CR30] Lechner M, Findeiss S, Steiner L, Marz M, Stadler PF, Prohaska SJ (2011). Proteinortho: detection of (co-)orthologs in large-scale analysis. BMC Bioinf.

[CR31] Lombard V, Golaconda Ramulu H, Drula E, Coutinho PM, Henrissat B (2014). The carbohydrate-active enzymes database (CAZy) in 2013. Nucleic Acids Res.

[CR32] Medema MH, Blin K, Cimermancic P, De Jager V, Zakrzewski P, Fischbach MA, Weber T, Takano E, Breitling R (2011) AntiSMASH: Rapid identification, annotation and analysis of secondary metabolite biosynthesis gene clusters in bacterial and fungal genome sequences. Nucleic Acids Res (Web Server issue):W339–W346 10.1093/nar/gkr466 PMC3125804 21672958

[CR33] Mulder KCL, Mulinari F, Franco OL, Soares MSF, Magalhaes BS, Parachin NS (2015). Lovastatin production: from molecular basis to industrial process optimization. Biotechnol Adv.

[CR34] Nowak RM, Jastrzębski JP, Kuśmirek W, Sałamatin R, Rydzanicz M, Sobczyk-Kopcioł A, Sulima-Celińska A, Paukszto Ł, Makowczenko KG, Płoski R, Tkach VV, Basałaj K, Młocicki D (2019). Hybrid de novo whole-genome assembly and annotation of the model tapeworm *Hymenolepis diminuta*. Sci Data.

[CR35] Ohashi H, Akiyama H, Nishikori K, Mochizuki J (1992). Asterric acid, a new endothelin binding inhibitor. J Antibiot (Tokyo).

[CR36] Okabe M, Lies D, Kanamasa S, Park EY (2009). Biotechnological production of itaconic acid and its biosynthesis in *Aspergillus terreus*. Appl Microbiol Biotechnol.

[CR37] Palanivel M, Mac Aogáin M, Purbojati RW, Uchida A, Aung NW, Lim SBY, Putra A, Drautz-Moses DI, Seaton S, Rogers TR, Schuster SC, Chotirmall SH (2020). Whole-genome sequencing of *Aspergillus terreus* species complex. Mycopathologia.

[CR38] Pastor FJ, Guarro J (2014). Treatment of *Aspergillus terreus* infections: a clinical problem not yet resolved. Int J Antimicrob Agents.

[CR39] Pritchard L, Glover RH, Humphris S, Elphinstone JG, Toth IK (2016). Genomics and taxonomy in diagnostics for food security: soft-rotting enterobacterial plant pathogens. Anal Methods.

[CR40] Rinderknecht H, Ward JL, Bergel F, Morrison AL (1947). Studies on antibiotics: 2. Bacteriological activity and possible mode of action of certain non-nitrogenous natural and synthetic antibiotics. Biochem J.

[CR41] Romsdahl J, Wang CCC (2019). Recent advances in the genome mining of *Aspergillus* secondary metabolites (covering 2012-2018). MedChemComm.

[CR42] Sato S, Okusa N, Ogawa A, Ikenoue T, Seki T, Tsuji T (2005). Identification and preliminary SAR studies of (+)-geodin as a glucose uptake stimulator for rat adipocytes. J Antibiot.

[CR43] Savitha J, Bhargavi SD, Praveen VK (2016). Complete genome sequence of soil fungus *Aspergillus terreus* (KM017963), a potent lovastatin producer. Genome Announc.

[CR44] Schliep KP (2011). phangorn: phylogenetic analysis in R. Bioinformatics..

[CR45] Seiboth B, Pakdaman BS, Hartl L, Kubicek CP (2007). Lactose metabolism in filamentous fungi: how to deal with an unknown substrate. Fungal Biol Rev.

[CR46] Sethi BK, Nanda PK, Sahoo S (2016). Characterization of biotechnologically relevant extracellular lipase produced by *Aspergillus terreus* NCFT 4269. 10. Braz J Microbiol.

[CR47] Shankar J, Tiwari S, Shishodia SK, Gangwar M, Hoda S, Thakur R, Vijayaraghavan P (2018). Molecular insights into development and virulence determinants of *Aspergilli*: a proteomic perspective. Front Cell Infect Microbiol.

[CR48] Shinohara C, Chikanishi T, Nakashima S, Hashimoto A, Hamanaka A, Endo A, Hasumi K (2000). Enhancement of fibrynolytic activity of vascular endothelial cells by chaetoglobosin A, crinipellin B, geodin and triticone B. J Antibiot.

[CR49] Sohail M, Ahmad A, Khan SA (2016). Production of cellulase from *Aspergillus terreus* MS105 on crude and commercially purified substrates. 3 Biotech.

[CR50] Stanke M, Waack S (2003) Gene prediction with a hidden Markov model and a new intron submodel. Bioinformatics 19:ii215–ii225 10.1093/bioinformatics/btg1080 14534192

[CR51] Steinbach WJ, Perfect JR, Schell WA, Walsh TJ, Benjamin DK (2004). In vitro analyses, animal models, and 60 clinical cases of invasive *Aspergillus terreus* infection. Antimicrob Agents Chemother.

[CR52] Takahashi H, Minami T, Okabe M, Park EY, Fujimoto T, Takahashi A, Murase M, Fukuyoshi S, Satou K, Kanamasa S (2020). Draft genome sequence of the *Aspergillus terreus* high-itaconic-acid-productivity strain IFO6365. Microbiol Resour Announc.

[CR53] Takatsuki A, Yamaguchi I, Tamura G, Misato T, Arima K (1969). Correlation between the anti-animal and anti-plant-virus activities of several antibiotics. (Studies on antiviral and antitumor antibiotics. XIX). J Antibiot (Tokyo).

[CR54] Thakur R, Shankar J (2017). Proteome profile of *Aspergillus terreus* conidia at germinating stage: identification of probable virulent factors and enzymes from mycotoxin pathways. Mycopathologia.

[CR55] Vinci VA, Hoerner TD, Coffman AD, Schimmel TG, Dabora RL, Kirpekar AC, Ruby CL, Stieber RW (1991). Mutants of a lovastatin-hyperproducing *Aspergillus terreus* deficient in the production of sulochrin. J Ind Microbiol.

[CR56] Vyas JM (2011). The duality of *Aspergillus terreus*: differential immune responses to distinct conidia. Virulence.

[CR57] Wilson K, Ausubel FM, Bent R, Kingston RE, Moore DD, Seidman JG, Smith JA, Struhl K (1987). Preparation of genomic DNA from bacteria. Current protocols in molecular biology pp.

[CR58] Wu S, Zhu Z, Fu L, Niu B, Li W (2011). WebMGA: a customizable web server for fast metagenomic sequence analysis. BMC Genomics.

[CR59] Zhang H, Yohe T, Huang L, Entwistle S, Wu P, Yang Z, Busk PK, Xu Y, Yin Y (2018). DbCAN2: A meta server for automated carbohydrate-active enzyme annotation. Nucleic Acids Res.

