# Supplementary material for: Complete genome sequence of lovastatin producer Aspergillus terreus ATCC 20542 and evaluation of genomic diversity among A. terreus strains
Source: Appl Microbiol Biotechnol. 2021 Jan 30;105(4):1615–27. doi: 10.1007/s00253-021-11133-0 (PMC7880949; doi:10.1007/s00253-021-11133-0)
Supplement: Supplementary file 1 — (PDF 324 kb) [file 253_2021_11133_MOESM1_ESM.pdf]

Applied Microbiology and Biotechnology

Complete genome sequence of lovastatin producer *Aspergillus terreus* ATCC 20542 and evaluation of genomic diversity among *A. terreus* strains

Małgorzata Ryngajłło[*1], Tomasz Boruta[2], Marcin Bizukojc[2]

1) Institute of Molecular and Industrial Biotechnology, Lodz University of Technology, B. Stefanowskiego 4/10, 90-924 Lodz, Poland
2) Faculty of Process and Environmental Engineering, Department of Bioprocess Engineering, Lodz University of Technology, ul. Wolczanska 213, 90-924 Lodz, Poland

*Corresponding author
Email: malgorzata.ryngajllo@p.lodz.pl
Telephone: +48 0426313354

Supplementary Tables

Table S1 Presence of orthologs of catabolic enzymes allowing utilization of carbohydrates based on sequence similarity with proteins of *A. niger* ATCC 1015 (employing threshold of 60% sequence identity across 60% of protein length).

Strain / Enzyme	NIH 2624	45A	M6925	w25	TN-484	IFO 6365	ATCC 20542	ATCC 20542 gene IDs
Glucose oxidase	4	3	4	3	2	2	3	HFD88_005098; HFD88_005956; HFD88_007298
Alpha-glucosidase	4	4	4	4	4	4	4	HFD88_001512; HFD88_003103; HFD88_006204; HFD88_007275
Beta-glucosidase	9	9	9	9	9	9	9	HFD88_000633; HFD88_006243; HFD88_000100; HFD88_002005; HFD88_008328; HFD88_005011; HFD88_010378; HFD88_010359; HFD88_001431
Beta-galactosidase	2	2	2	2	2	2	2	HFD88_000677; HFD88_006315
Galactokinase	1	1	1	1	1	1	1	HFD88_009871
Galactose-1-phosphate uridylyltransferase	1	1	1	1	1	1	1	HFD88_002772
UDP-glucose 4-epimerase	1	1	1	1	1	1	1	HFD88_004936
UDP-glucose pyrophophorylase	1	1	1	1	1	1	1	HFD88_004565
Phosphoglucomutase	2	2	2	2	2	2	2	HFD88_004565
Invertase	2	1	1	2	2	2	2	HFD88_000821; HFD88_004725
Hexokinase	3	3	3	3	3	3	3	HFD88_010162; HFD88_003424; HFD88_008047
Mannose-6-phosphate isomerase	2	2	2	2	2	2	2	HFD88_006399; HFD88_000452
Oxaloacetate acetylhydrolase	2	2	2	2	2	2	2	HFD88_009453; HFD88_010407
Malate synthase	3	3	3	3	2	3	3	HFD88_003886; HFD88_004697; HFD88_002011
Isocitrate lyase	2	2	2	2	2	2	2	HFD88_003886; HFD88_004697
L-Arabitol dehydrogenase	2	2	2	2	2	2	2	HFD88_000634; HFD88_003751
D-xylose reductase	1	1	1	1	1	1	1	HFD88_008228
L-sylulose reductase	4	4	4	4	4	4	4	HFD88_003695; HFD88_008283; HFD88_001090; HFD88_008006
D-xylulose reductase	4	3	4	3	4	3	3	HFD88_008288; HFD88_009120; HFD88_004639; HFD88_005020

Aldehyde reductase	4	4	4	4	4	4	4	HFD88_001875; HFD88_003199; HFD88_008111; HFD88_000427
D-xylulose kinase	1	1	1	1	1	1	1	HFD88_003734

Table S2 Presence of orthologs of catabolic enzymes associated with organic acid production based on sequence similarity with proteins of *A. niger* ATCC 1015 (employing threshold of 60% sequence identity across 60% of protein length).

Strain / Enzyme	NIH 2624	45A	M6925	w25	TN-484	IFO 6365	ATCC 20542	ATCC 20542 gene IDs
Pyruvate dehydrogenase E1 component subunit beta	2	2	2	2	2	2	2	HFD88_004098; HFD88_008785
Pyruvate dehydrogenase E1 component subunit alpha	1	1	1	1	1	1	1	HFD88_010188
Pyruvate dehydrogenase E2 component	1	1	1	1	1	1	1	HFD88_010188
Citrate synthase	2	2	2	2	2	2	2	HFD88_002002; HFD88_006968
Aconitate hydratase	2	2	2	2	2	1	2	HFD88_001544; HFD88_001145
Isocitrate dehydrogenase (NAD+) subunit	2	2	2	2	2	2	2	HFD88_004656; HFD88_006462
Isocitrate dehydrogenase [NAD] subunit 2	2	2	2	2	2	2	2	HFD88_006462; HFD88_004656
2-oxoglutarate dehydrogenase	2	2	2	2	2	2	2	HFD88_004285; HFD88_009432
Dihydrolipoyllysine-residue succinyltransferase	1	1	1	1	1	1	1	HFD88_005618
Succinyl-CoA ligase [GDP-forming] subunit alpha	2	2	2	2	2	2	2	HFD88_010271; HFD88_006662
Succinyl-CoA ligase [GDP-forming] subunit beta	1	2	1	1	1	1	1	HFD88_008362
Succinate dehydrogenase [ubiquinone] flavoprotein subunit	1	1	1	1	1	1	1	HFD88_003060
Fumarate hydratase	1	1	1	1	1	1	1	HFD88_002093
Oxaloacetate acetylhydrolase	3	3	3	3	3	3	3	HFD88_010407; HFD88_009453; HFD88_005712
L-lactate dehydrogenase	1	1	1	1	1	1	1	HFD88_007813
Citramalate synthase	1	1	1	1	1	1	1	HFD88_006027
Isopropylmalate isomerase (IPMI)	1	1	1	1	1	1	1	HFD88_002799
Isocitrate lyase	2	2	2	2	2	2	2	HFD88_001243; HFD88_002050

Malate synthase	1	1	1	1	1	1	1	HFD88_002011
Glucose oxidase	3	2	2	3	1	1	2	HFD88_005956; HFD88_005098
Pyruvate carboxylase	1	1	1	1	1	1	1	HFD88_000372
Malate dehydrogenase	2	2	2	2	2	2	2	HFD88_008419; HFD88_003846

Table S3 Unique genes of *A. terreus* ATCC 20542 with KEGG annotation.

ATCC 20542 gene ID	KEGG ID	Description
HFD88_000052	K23272	Pyranose oxidase [EC:1.1.3.10]
HFD88_000053	K21899	Aldos-2-ulose dehydratase [EC:4.2.1.110]
HFD88_001689	K16219	Protein N-terminal methyltransferase [EC:2.1.1.244]
HFD88_003313	K03781	Catalase [EC:1.11.1.6]
HFD88_003314	K00844	Hexokinase [EC:2.7.1.1]
HFD88_006108	K05665	ATP-binding cassette, subfamily C (CFTR/MRP), member 1 [EC:7.6.2.3]
HFD88_008996	K01426	Amidase [EC:3.5.1.4]
HFD88_009056	K17069	O-acetylhomoserine/O-acetylserine sulfhydrylase [EC:2.5.1.49 2.5.1.47]
HFD88_010689	K00069	15-hydroxyprostaglandin dehydrogenase (NAD) [EC:1.1.1.141]
HFD88_010691	K03380	Phenol 2-monooxygenase (NADPH) [EC:1.14.13.7]

Table S4 Conservation of genes encoding key virulent factors in *A. terreus* reported by Thakur and Shankar, 2017. Conservation of genes in *A. terreus* genomes is based on proteinortho results.

Gene name	Description	NIH 2624 gene ID	ATCC 20542 gene ID	Conservation in *A. terreus* genomes
-	Terrelysin	ATEG_03556	HFD88_000914	Conserved in all
hog1	Mitogen-activated protein kinase	ATEG_00489	HFD88_006440	Conserved in all
mpkC	Mitogen-activated protein kinase	ATEG_06557	HFD88_003645	Conserved in all
cgrA	rRNA-processing protein	ATEG_10388	HFD88_000168	Conserved in all
creB	Ubiquitin carboxyl-terminal hydrolase	ATEG_03173	HFD88_001301	Conserved in all
pepP	Xaa-Pro aminopeptidase	ATEG_06859	HFD88_001743	Conserved in all
mep	Metalloproteinase	ATEG_07544	HFD88_004788	Conserved in all
myoA	Myosin-1 OS	ATEG_07759	HFD88_005016	Conserved in all

Supplementary Figures

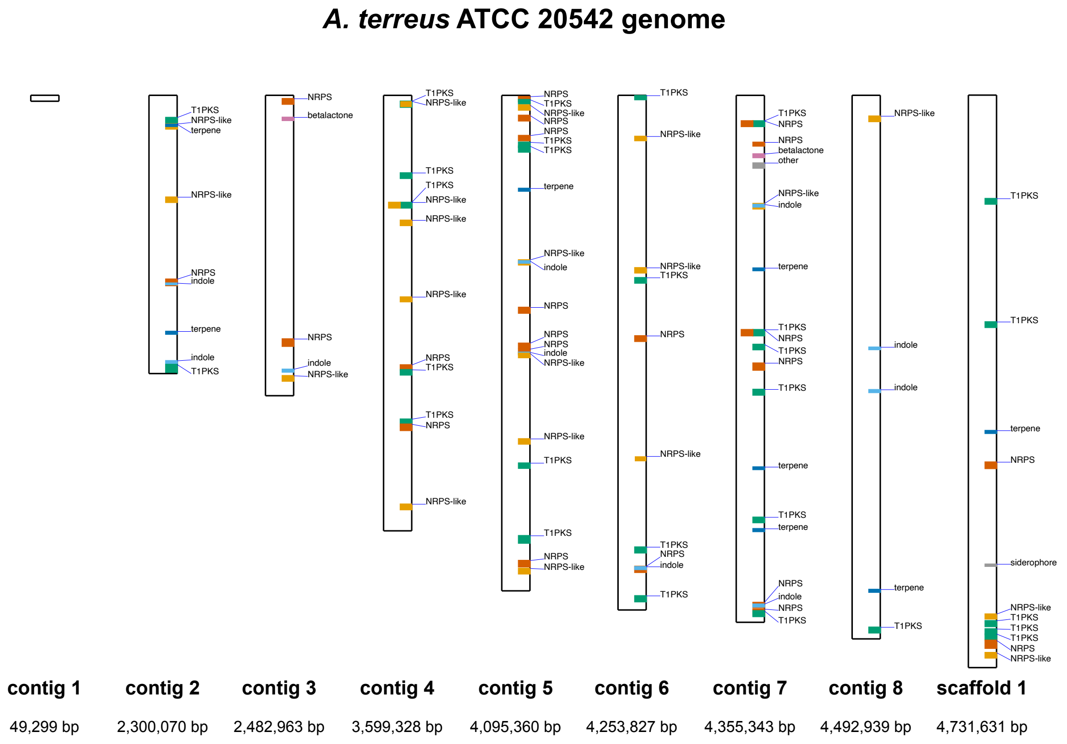

Fig. S1 Map of *A. terreus* ATCC 20542 genome with marked SM clusters predicted by AntiSMASH

References

Thakur R, Shankar J (2017) Proteome profile of *Aspergillus terreus* conidia at germinating stage: identification of probable virulent factors and enzymes from mycotoxin pathways. Mycopathologia 182:771-784
